# Patient and caregiver experiences of palliative care co-management in oncology

**DOI:** 10.1017/S1478951524001846

**Published:** 2025-01-13

**Authors:** Joanna Veazey Brooks, Taynara Formagini, Claire Poague, Christian T. Sinclair, Karin Porter-Williamson

**Affiliations:** 1 Department of Population Health, University of Kansas School of Medicine, Kansas City, KS, USA; 2 University of Kansas Cancer Center, Kansas City, KS, USA; 3 Division of Palliative Medicine, University of Kansas School of Medicine, Kansas City, KS, USA; 4 Department of Family Medicine, University of California, San Diego, CA, USA

**Keywords:** Outpatient palliative care, palliative care, value-aligned care, patient-centered medicine, palliative care co-management

## Abstract

**Objectives:**

Historically, patients with cancer were referred to palliative care near the end of life. In recent years, the increased integration of palliative care throughout the entire trajectory of illness has helped patients with cancer better manage their symptoms and improve QOL. However, it is unknown how patients think about the presence and role of earlier, integrated palliative care. This study explored how patients and caregivers experience cancer care in the context of palliative care co-management with oncology.

**Methods:**

We conducted interviews with 18 patients and 13 caregivers to investigate perspectives, attitudes, and experiences surrounding cancer care, specifically with their experiences of co-management with a palliative care outpatient clinic and oncology. Using grounded theory, we identified a typology of patient and caregiver approaches when discussing the care they received and/or desired.

**Results:**

Our data revealed 3 approaches to thinking about palliative care in cancer care. While some participants embraced the “Cure Centrality” approach, caring only about fighting the disease, others adopted a “Quality-of-Life (QOL) Centrality” approach, desiring their health-care team to prioritize a broader range of concerns. A third approach, The “Dual Centrality” approach, espoused values from both approaches.

**Significance of results:**

While co-management of palliative care and oncology is complementary by design, our data suggest that patients and caregivers take a variety of approaches to their copresence. For some patients, palliative care served as an important legitimizing resource for patients desiring expanded priorities in their care (e.g. higher value on QOL and symptom management) and enabling patient-centered care.

## Introduction

In 1971, President Nixon declared a “War on Cancer” with the National Cancer Act (Alam 2021). Military metaphors have dominated narratives around cancer throughout the mid-20th century (Ellis et al. 2015; Mrig and Spencer 2018), and in this common cultural model, the emblematic cancer patient “fights cancer” and endures treatment in order to reach victory: cure (Harrington 2012). This singular focus on eliminating cancer inherently demotes quality-of-life (QOL) considerations to secondary importance and creates a falsely dichotomous narrative of fight-win-live vs quit-lose-die.

In recent years, several changes in oncology care have begun to challenge this traditional model. First, advances in cancer treatment are extending life expectancies for individuals with cancer (Miller et al. 2019). Next, there is increased recognition that patients exert power in shaping their illness and health-care experiences, and patient preferences are receiving more attention given their increased ability to access information and seek opinions from other physicians as consumers in a market (Bardes 2012; Boyer and Lutfey 2010; Ebeling 2011; Hafferty and Light 1995; Vinson 2016).

Concurrently, the field of supportive care in cancer is growing, including services such as counseling, nutrition, and palliative care (Institute of Medicine and National Research Council 2006). Palliative care is increasingly offered throughout the cancer journey, including during active treatment and initial stages of the disease, driven by evidence that it improves QOL and addresses symptoms from both treatment and disease (Bakitas et al. 2009; Dumanovsky et al. 2016; Howie and Peppercorn 2013; Hui et al. 2010; Huo et al. 2021; Temel et al. 2010). In the recent decade, an expansion from inpatient palliative care to palliative care clinics in outpatient settings have also enabled longitudinal palliative care co-management (Hui et al. 2020). Studies show palliative care can improve outcomes (Howie and Peppercorn 2013; Rabow et al. 2004; Vanbutsele et al. 2018), and when integrated early in the treatment for some cancers it may extend life expectancy (Bakitas et al. 2009; Bruera and Hui 2010; Temel et al. 2010).

In this changing clinical context where specialty palliative care is present as a co-manager alongside oncology care (and not restricted to end-of-life), it is unclear how palliative care may impact the way patients and caregivers make sense of their illness journey. Other scholars have considered the role and values of palliative care at the end-of-life, when patients are choosing to end “curative” therapy and pursue hospice (Livne 2019; Mrig and Spencer 2018). However, little is known about how earlier, integrated palliative care offered as a co-existing service *with* oncology impacts patients’ experiences of care. In this changed structure, the values of palliative care, coupled with the growing emphasis on patient agency, are likely to provide counterpressure to the all-encompassing traditional battle metaphor in cancer care. In this paper, we explore how patients and caregivers experience cancer care in the context of receiving palliative care while in treatment, and navigate between the traditional “War on Cancer” approach and alternative approaches.

## Methods

### Study design and setting

This study is part of a larger qualitative interview project designed to investigate the perspectives, attitudes, and experiences of patients, caregivers, and clinicians about cancer care, palliative care, and symptom communication (Brooks et al. 2020; Formagini et al. 2022). Previous papers from this project have addressed separate and distinct research questions. In this paper, we followed a grounded theory approach to explore patients and caregivers experiences in making sense of their disease, treatment, and illness journey while receiving oncology and palliative care concomitantly (Glaser and Strauss 1999). The project was conducted at an outpatient cancer center affiliated with an academic medical center with an established palliative care program. The palliative care clinic at our health system is a mature, multidisciplinary clinic that is well-integrated into the bone marrow transplant and oncology clinics with over 20 half-day sessions each week making nearly 3,000 visits per year. Referrals are primarily based on oncologists’ perception of need or patient self-referral; triggers have not been widely utilized. Participants in our study did not share the same oncologist. While we did not specifically track which oncologist they had, there are over 100 who are in practice at our cancer center. In the outpatient setting, our palliative care team includes physicians, nurse practitioners, social workers, and nurses. The palliative care clinics follow a continuity model where patients keep the same clinic team. There are 6 physicians and 2 nurse practitioners staffing clinics. The project received ethical approval from the University of Kansas Medical Center IRB.

### Participants and recruitment

Participants included adult patients receiving care for solid organ tumors, hematologic malignancies, and blood and marrow transplants for cancer and other blood disorders, and their caregivers. Because we wanted to capture a multitude of experiences with palliative care, participants with different levels of exposure to palliative care were purposefully recruited using theoretical purposive sampling. This includes patients and caregivers at both initial and follow-up appointments, as well as a few “disconfirming cases” (1 patient and 1 caregiver who did not receive palliative care, and 2 bereaved caregivers).

We partnered with the cancer center’s outpatient palliative care team and other cancer center staff to identify patients and caregivers willing to participate in interviews. Potential participants were introduced to the study by a nurse, and if interested, an appointment with a study team member was conducted to obtain informed consent and conduct the interview. The nurses made real-time evaluations about which patients to invite, taking the health status of patients into consideration. Some individuals declined to participate due to scheduling constraints or lack of interest, but we cannot specify how many individuals were approached. One individual consented to participate but later decided not to start the interview.

An interview guide was used to prompt participants to elaborate on their experiences and perspectives of the care they were receiving, including experiences with palliative care. The guide was developed by the first author in collaboration with palliative care clinicians. A total of 18 patients and 13 caregivers completed the interviews. Interviews were conducted at the cancer center, except for 1 caregiver who preferred to have a phone interview. Participants were not required to be part of a patient–caregiver dyad to complete the interview, and in general, we only interviewed 1 member of the patient–caregiver pair. Participants were often interviewed separately, but occasionally, caregivers were also present in the room during patient interviews. Interviews ranged from 14 to 53 minutes and were conducted between June and December of 2018. All participants were offered a $30 gift card.

### Data analysis

We used principles of grounded theory to conduct the data analysis (Charmaz 2006). The interview recordings were professionally transcribed, blinded, and verified by the authors to ensure accuracy. Next, we open-coded a subset of 4 transcripts (2 patients and 2 caregivers) to document preliminary codes (Charmaz 2006). This procedure was conducted by 3 authors independently, and codes and changes were documented to create an audit trail. We developed a codebook with all codes identified from the data together with a description of the code, and examples of quotes. Subsequently, all transcripts were coded by 1 author and reviewed by a second author. The research team then held a series of meetings to review the concepts and resolve questions and discordances. The coding process was completed using NVivo 11 qualitative software.

The analysis drew primarily on data from 2 codes: “palliative care versus oncology” and “patient values/quality of life” which were the most relevant for the understanding of participants’ experiences in making sense of their disease, active treatment, and illness journey while receiving oncology and palliative care concurrently. Open and axial coding, and iterative discussions uncovered the identification of 3 different approaches to cancer care, reported here (Charmaz 2006). Throughout the paper, participant quotes are presented with a random identifier.

## Results

The data collected from patients and caregivers contained information not only regarding the differences between the type of care provided by oncologists and palliative care physicians, but also gave light to different approaches that participants used to think about the care they were receiving and to communicate varying preferences and priorities. We organize the findings by approach: (1) Cure Centrality, (2) QOL Centrality, and (3) Dual Centrality. In our data, approaches were not patterned by role (patient or caregiver).

### Cure Centrality

Consistent with prior literature about battle metaphors in cancer care (Mrig and Spencer 2018), respondents who adopted the Cure Centrality approach typically perceived everything besides beating cancer as “peripheral.” Here, participants commonly elucidated their oncologist as the central provider in their care.

… and I want [oncologist] to keep being able to do [my disease and treatments]. I don’t want him to be distracted with some of the other peripheral stuff because that would take away from his ability to maybe find a cure or find a treatment that would actually work better for me […]. (Participant 59)

Participants communicating this approach also identified what they expected from oncologists and palliative care physicians. They often saw these roles as completely distinct:

I wouldn’t talk to palliative care about my oncology or my care with oncology, just like I don’t think I would talk to my oncology team about what I speak to palliative care about. […], I don’t think I would call a football coach to talk about baseball, you know? I would kind of talk to a baseball coach about baseball and a football coach as far as football is concerned. (Participant 93)

Here, there were clear and rigid delineations around the scope of oncology and palliative care, to such a degree that Participant 93 describes them as different “sports.” It was common for participants in this group to show a high level of esteem for oncologists, seen as “super experts,” who were too busy to focus on anything besides the cancer:

*But mostly I’m listening to what [oncologist]’s got to say because [oncologist] is the super expert. I consider [oncologist] a super expert.* Interviewer: And what about the palliative care team? Would you say they’re super expert in a different area or do you think they’re less super expert? *Participant: I think they’re really good in their area.* (Participant 11)

Overall, participants who adopted Cure Centrality viewed curing the cancer as the highest priority. Among these patients, palliative care had a clear peripheral role, with QOL and symptom management rarely discussed.

### QOL Centrality

Participants embracing QOL Centrality identified shortcomings that stemmed from the singular focus on disease found in Cure Centrality, and often articulated a belief that symptom management and QOL were integral to what they wanted in cancer care. Participants sometimes felt dismissed when bringing up symptoms or QOL concerns with the oncologist because they were told that symptoms were “part of the disease”:

I think that pain is a specific example. So I’ve brought it up to my oncology team and they kind of say, “Well, maybe that’s your new normal.” And […] I’ve also told them that I’m just so tired all the time. And so they tell me to decrease my methadone, which would increase my pain. And I say but I’m also in pain. And they’re saying, “Well, maybe that’s your new normal” type thing. (Participant 25)

Basically every time I’d ask my oncologist, “Yes, you’re just going to have to have that pain.” That was the answer. It’s rib pain, and that is a normal thing for myeloma. That’s just the way it is. […] “that’s just part of it, that’s part of the disease.” And yeah, it’s part of the disease to get it, but it isn’t necessarily part of the disease to keep it. So I think palliative care is looking for other avenues. (Participant 99)

Patients in this group actively questioned the messages they received to endure anything without question. For example, Patient 99 showed an ability to dismantle the logic behind those messages by critically questioning whether “getting” a symptom really necessitates “keeping it.” Within QOL Centrality, there is no inherent need to suffer or to automatically accept a “new normal” with lower QOL. Instead, seeking assistance to decrease symptom burden and improve QOL was highly valued.

Individuals employing QOL Centrality talked about how palliative care was able to complement oncology to address what was “missing” from the oncologists.

Just like each thing that’s wrong, like figuring out something for my joints, or my nausea, sleeping. Instead of just putting a Band-Aid on it or just missing it, which is generally what’s been happening. […] Whereas palliative care’s response for the same problem would be “oh well, let’s figure out why and how to fix it.” Like what’s the root cause of it. (Participant 81)

Participants also recognized that palliative care considered different data inputs than oncology:

My oncologists do it from a numbers perspective. They look a lot at my blood work and all that kind of stuff. Whereas palliative does it from a how are you feeling perspective […] I much more appreciate the how are you feeling perspective […] You have to do something to get your numbers up and that kind of stuff […] but there’s certain things that don’t show up in your numbers that they can’t tell that you’re in pain by a blood test. (Participant 25)

As seen in this quote, participants adopting QOL Centrality resisted being reduced to their disease or lab values. Several interviewees explained how palliative care had specifically helped them expand their care to align with personal values:

All we’ve ever dealt with is simply the clinical. We’ve never dealt with anything that is outside that other than when we go home and then we talk about well, can we get an Amtrak trip into this, how can we do this or that and work around going to the doctor, to the clinical doctor. So this is the first time [with palliative care] that the picture’s gotten broader. (Participant 53)

In contrast to Cure Centrality, participants who embraced the QOL Centrality saw QOL as centrally important and voiced appreciation for the additional focus the palliative care team provided.

### Dual Centrality

The Dual Centrality approach expressed values found in both former groups. This third group, similar to QOL Centrality, explained the importance of receiving care that goes beyond an exclusive focus on disesase. However, this group also quickly pivoted to explain the oncologist’s specialized focus. For example, the caregiver below goes back and forth between justifying oncology’s approach and explaining the benefits of palliative care:

And they [oncologist team] weren’t bad […] but I just felt like when I come to palliative they’re more listening to us, and compassionate, wanting to make her comfortable. Just more concerned. Not that the other people aren’t, because they’re basically just going off of what her labs are, what’s going on with her body. But here I felt like they’re trying to make her comfortable … (Participant 96)

Several participants also talked about the difference in time between the 2 specialties.

… we love the oncologists down there. But as you know, the oncologists are very busy, so they don’t sit and talk to you as a person very long. […] they very seldom ever actually talk to you for very long because they’re in a hurry and they’ve got a lot of patients. So I think [wife] really enjoyed having a [Palliative Care] doctor sit and visit with her about what she was going through. (Participant 54)

In addition to time, this group also noted oncologists’ narrow focus, much like the respondents with QOL Centrality, but were quick to neutralize the potential shortcoming:

Sometimes I think the oncologists are tunnel visioned a little bit. And that’s not a bad thing. But his surgical oncologist, great guy, we love him. But a lot of times [Patient] would say I’m tired or I’m whatever, whatever, and it was still just about the cancer and the surgery and the tumor. […], that’s where I kind of feel like palliative care is a good addition because they may have ideas that your oncology team won’t have. (Participant 77)

Overall, participants in this group saw the complentary strengths of both teams.

## Discussion

We investigated the experience of co-management between oncology and palliative care for patients with cancer and their caregivers. We explored how 31 participants made sense of their care in a structure that included both oncology and palliative care. We found evidence for 3 approaches in our study: Cure Centrality, QOL Centrality, and Dual Centrality.

Participants in the 3 groups presented different perspectives in relation to their goals of cancer care, core values, expectations, and experiences with their health-care team, a finding consistent with prior research about the use of different metaphors in cancer care (Semino et al. 2018). Patients and caregivers conveying Cure Centrality viewed getting rid of cancer as the clear top priority – all other concerns were delegated to secondary importance. Participants with a Dual Centrality approach fluctuated, placing a high value on cure while also describing a desired approach to cancer care that included concerns such as QOL, symptom control, and plans for the future. An earlier study previously found that individuals can use “contradictory metaphors” when describing their experiences with cancer (Gibbs and Franks 2002). Finally, participants adopting the QOL Centrality were typically dissatisfied with the inattention from oncology to their QOL and resisted the idea that their “new normal” had to include a high symptom burden or a dismissal of all other life priorities.

Notably, palliative care seems to provide patients and caregivers with a level of support that helps them disengage from the classic cancer “battle” narrative, in which the patient dutifully suffers through symptoms. Palliative care has already been shown to provide improved symptom management and QOL, even during active treatment (Bakitas et al. 2009; Hawley 2017; Howie and Peppercorn 2013; Temel et al. 2010). Equally important, however, is that our data suggest palliative care can help show patients that it is reasonable to prioritize and address symptoms and QOL when treating cancer. In a complex and changing health-care environment, the growing presence of palliative care as a specialty of medicine embedded in cancer care that is focused on areas besides “beating the cancer” seems to provide structural legitimacy for patient and caregiver approaches that include other values alongside the hope for cure. Our study shows that patients and caregivers are indeed actively choosing, from within their current structure of care, different approaches for how they make sense of their care and health-care teams.

This study has limitations. First, it took place at one outpatient cancer center with an outpatient palliative care clinic and team and our findings may not be transferable to other cancer center settings, especially those with less access to palliative care. Next, we are unable to assess whether patients that are not exposed to palliative care have substantially different approaches to cancer care. It could be that patients not exposed to palliative care would be even more likely to demonstrate Cure Centrality, suggesting that this appoach may be more prevalent in the overall cancer patient population than our data would suggest. While our study is unable to assess whether participants already had a QOL Centrality approach prior to palliative care, those with unsatisfactory oncology care experiences with communication around symptoms seemed particularly enthusiastic about the QOL Centrality. It is possible that patients that were closer to end of life were more likely to favor the QOL Centrality; however, our study included a mix of patients in different stages of disease and treatment and no clear patterns were found. Future studies could stratify patients by stage of the disease and treatment to assess whether those impact approaches. As the Dual Centrality approach makes clear, some participants saw the benefits of both approaches, elevating cure and QOL. Longitudinal studies could assess whether those in this group eventually migrate more fully to Cure or QOL Centrality or whether they remain persuaded by the values of both. Finally, while we did not collect sociodemographic data on our participants, future studies should consider the relationship between patient demographics (e.g. education, gender, race, and rurality) and approach to identity any patterns and disparities.

## Conclusions and practice implications

Cancer care delivery is in a state of transition, as supportive services for patients and caregivers expand and as the broader patient-centered care movement impacts the delivery of health care. Our data suggest that some patients and caregivers still desire care focused only on beating cancer – an approach that may in fact be well-aligned with their values. Others, however, are moving beyond what they consider an overly narrow focus on just *fighting* cancer, and are also embracing the centrality of symptoms, QOL, and their own personal values and goals. Health-care providers and leaders should recognize and strategically consider how structural changes to health care, such as the development and physical copresence of specialty palliative medicine clinics, can serve as legitimizing resources for patients, augmenting the approaches to care that are accessible to them. Moving toward patient-centered care may require that these legitimizing resources are actually built into the structure and locality of care, and that health-care teams help patients understand the complentarity between the specialities. Future research could explore analogies and scripts used by these 2 teams to help patients understand and navigate co-management between oncology and palliative care. As we strive to improve health outcomes as well as health-care delivery experiences, empowering patients to truly decide what they want for their care and from their health-care team, and how they want to embody the role of a patient with cancer, are essential steps to providing high-quality, patient-centered care.
